# 4D Doppler Ultrasound in High Grade Serous Ovarian Cancer Vascularity Evaluation—Preliminary Study

**DOI:** 10.3390/diagnostics11040582

**Published:** 2021-03-24

**Authors:** Marek Jerzy Kudla, Michal Zikan, Daniela Fischerova, Mateusz Stolecki, Juan Luis Alcazar

**Affiliations:** 1 Department of Perinatology and Oncological Gynecology, Faculty of Medical Sciences, Medical University of Silesia, Medyków 14, 40-752 Katowice, Poland; 2 Department of Gynecology and Obstetrics, Charles University—First Faculty of Medicine and Bulovka University Hospital, Budinova 67/2, 181 00 Prague, Czech Republic; michal.zikan@lf1.cuni.cz; 3 Gynecologic Oncology Centre, Department of Obstetrics and Gynecology, First Faculty of Medicine, Charles University, General University Hospital, Apolinarska 18, 12851 Prague, Czech Republic; daniela.fischerova@seznam.cz; 4 Department of Gynecology, Obstetrics and Oncological Gynecology, Faculty of Medical Sciences, Medical University of Silesia, Batorego 15, 41-902 Bytom, Poland; mj.stolecki@gmail.com; 5 Department of Obstetrics and Gynecology, Clínica Universidad de Navarra, University of Navarra, Avenida Pio XII, 36, 31008 Pamplona, Spain; jlalcazar@unav.es

**Keywords:** 4D ultrasound, Doppler, STIC, ovary, cancer

## Abstract

The aim of the study was to evaluate the usefulness of 4D Power Doppler tissue evaluation to discriminate between normal ovaries and ovarian cancer tumors. This was a prospective observational study. Twenty-three cases of surgically confirmed ovarian High Grade Serous Carcinoma (HGSC) were analyzed. The control group consisted of 23 healthy patients, each matching their study-group counterpart age wise (±3 years) and according to their menopausal status. Transvaginal Doppler 4D ultrasound scans were done on every patient and analyzed with 3D/4D software. Two 4D indices—volumetric Systolic/Diastolic Index (vS/D) and volumetric Pulsatility Index (vPI)—were calculated. To keep results standardized and due to technical limitations, virtual 1cc spherical tissue samples taken from the part with highest vascularization as detected by bi-directional Power Doppler were analyzed for both groups of ovaries. Values of volumetric S/D indices and volumetric PI indices were statistically lower in ovarian malignant tumors compared to normal ovaries: 1.096 vs. 1.794 and 0.092 vs. 0.558, respectively (*p* < 0.001). The 4D bi-directional Power Doppler vascular indices were statistically different between malignant tumors and normal ovaries. These findings could support the rationale for future studies for assessing this technology to discriminate between malignant and benign tumors.

## 1. Introduction

Blood supply and the net of blood vessels play a key role in rapidly growing neoplasm tumors. This issue has been examined extensively with the use of 2D and 3D ultrasound techniques combined with color/power Doppler and spectral Doppler techniques [1,2,3,4]. The 3D Doppler (so called “3D angiography”), in spite of limitations [5,6], already proved its usefulness in differentiating between normal and malignant ovaries [4,7]. The method was modified then to achieve better standardization and universality [8]. Still, even with combined 2D and 3D methods and highly experienced ultrasonographers involved, in about 10% of cases, the nature of the tumor cannot be properly diagnosed [9,10]. The advent of 4D angiography (4D visualization combined with power Doppler options) together with the option called Spatio-Temporal Image Correlation (STIC) has allowed for detailed estimation of blood flow changes in the tissue under investigation within one cardiac cycle. It has paved the way for using 4D angiography in differentiating pathological tissues. Using this technology, new volumetric vascular indices: volumetric Systolic/Diastolic Index (vS/D) and volumetric Pulsatility Index (vPI), have been developed [11,12].

The objective of this study was to assess whether volumetric 4D indices are different when derived from malignant tissue vessels as compared to normal tissue vessels. For this purpose, we compared volumetric 4D indices obtained from a network of vessels from High Grade Serous Carcinoma (HGSC) tumors with ovarian stromal vessels from normal ovaries.

However, this is a proof-of-concept study and we evaluated the ability of the technical method (STIC) to distinguish between normal and pathological vessels in order to recognize malignancy. To avoid additional factors influencing tumor vascularity, we choose the dichotomic set—HGSC versus normal tissue—as a model.

## 2. Materials and Methods

This is a prospective observational study performed at three gynecological oncological centers (Clinical Department of Oncological Gynecology, Medical University of Silesia, Katowice, Poland; Gynecological Oncology Centre, Charles University in Prague, Czech Republic; and in Department of Obstetrics and Gynecology, University of Navarra, Pamplona, Spain). A non-consecutive series of women with suspicious adnexal masses were recruited at any of the three participating centers.

The inclusion criteria for the study group were the presence of a highly suspicious adnexal mass and no medical problems causing irregular heart rate. The suspicious adnexal mass was classified using subjective assessment by an experienced examiner and should contain either a solid component with vascularization within the uni-/multilocular-solid mass or being characterized by a vascularized solid tumor (color score 3 or 4 using IOTA (International Ovarian Tumor Analysis) terminology) [13]. Additionally, the distance between the active face of the transvaginal transducer head and surface of the tumor (the vascularized region) had to be smaller than or equal to 20 mm.

Patients with neoadjuvant chemotherapy for ovarian cancer were excluded. Additional exclusion criteria were as follows: non-malignant histology of the tumor, malignant histology other than HGSC, and no histologic diagnosis available.

For each patient included, we recruited a control patient. All patients for the control group were recruited from the Outpatient Unit at the Clinical Department of Oncological Gynecology, Medical University of Silesia, Katowice, Poland, based on a routine check-up for cervical cancer screening. The Outpatient Unit provides medical care services for the public from the whole region, not only for oncological purposes. All of the patients were asked to participate after the nature of the study was fully explained by one of the authors (M.J.K.).

Control group inclusion criteria were as follows: healthy asymptomatic women with no medical problems causing irregular heart rate, attending routine check-up, ovaries with no visible pathologies like simple cysts, dermoids or endometrial cysts, ovaries of typical size enabling total volume of the ovary to be recorded in a single 4D (STIC) sweep as well as in a 3D scan, and no hormonal gynecologic treatment in at least two months prior to the examination.

All of the premenopausal ovulating women were examined in the early follicular phase of the cycle. As in the study group, the distance between the active face of the transvaginal transducer head and the ovary surface had to be smaller than or equal to 20 mm.

All of the patients gave oral informed consent to participate after the nature of the study was explained. The study was approved by local Institutional Review Boards in every participating institution (IRB approval ID: KNW/0022/KB/42-1/14).

The cases of High Grade Serous Carcinoma (HGSC) and control cases were analyzed, which were confirmed by post-surgery histopathological examination. We decided to focus on one histotype only to exclude a potential heterogeneity of vascularization across different histotypes. Moreover, High Grade Serous Carcinoma is the most frequent type of ovarian cancer.

All three centers were using the same ultrasound systems: Voluson E8 Expert (GE Medical, Zipf, Austria) with the same set of transducers. The transducer type used in this study was the endovaginal RIC 5-9-D volumetric endocavitary probe (GE Healthcare, Zipf, Austria) with a frequency range of 5–9 MHz. All three systems were equipped with the Spatio-Temporal Image Correlation (STIC) option. The Doppler option used in the study was the bi-directional Power Doppler, the so-called High Definition Flow (HDF).

Prior to starting the study, identical settings to be applied during the record collection were maintained in all three systems used in the participating hospitals. The HDF Doppler settings were set as follows: depth of the HDF window 42 mm, PRF 0.6 kHz, gain-1, frequency “mid,” HDF quality “high,” WMF “low 1,” smooth 3/5, ensemble 10, flow resolution “high,” line density 8, balance “200,” HD map “1,” low filter 3. The STIC sweep angle was 90 degrees and acquisition time 15 s.

All of the ultrasonographically analyzed ovarian tumors had visible vascularization within their volumes.

For every patient recruited to the study group, on top of routine ultrasound preoperative scans, an additional 4D HDF (STIC) scan of the tumor was done. In some of the cases when single scan visualization of the whole tumor was difficult, we accepted and recorded the most vascularized solid component closest to the transvaginal probe. Records were stored in the memory of the system. As stated above, after this, a control case was recruited for such a patient.

In the sub-set of post-menopausal controls, the ovary of better visibility was recorded. In the sub-set of premenopausal patients within the control group, the volume from the non-ovulating ovary during early follicular phase of the menstruation cycle was recorded.

Four-dimensional STIC-HDF calculations were done on a personal computer, with 4D-View version 10.5 BT12 software (GE Healthcare, Zipf, Austria). Virtual spherical sampling was decided to be used as a method to calculate vascularization indices for the volume—the reliability of which was positively confirmed in previous 3D and 4D studies [12,14,15]. The methodology of 4D STIC data processing is presented on Supplementary Video S1.

**Figure 1:**
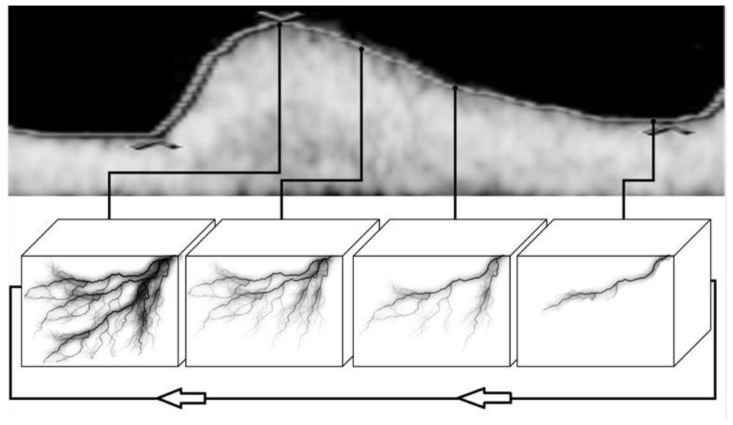
Schematic principle of creating a Spatio-Temporal Image Correlation (STIC) 4D record. A 4D record consists of approximately eleven 3D records (volumes) that correspond to the various phases of vascular changes during one heart cycle and that, when presented one by one, form a moving spatial image. The figure shows only four representative 3D records for simplicity.

The first step in calculations for every record was to activate a 4D sequence in order to work with a sequence of 3D HDF volumes forming the STIC loop (covering the time of one heart cycle) (Figure 1).

After magnification and bringing the point of highest vascularization of the STIC (4D) record to the center of the screen, each volume in the sequence was saved as a 3D volume/record with a different, consecutive number given.

**Figure 2:**
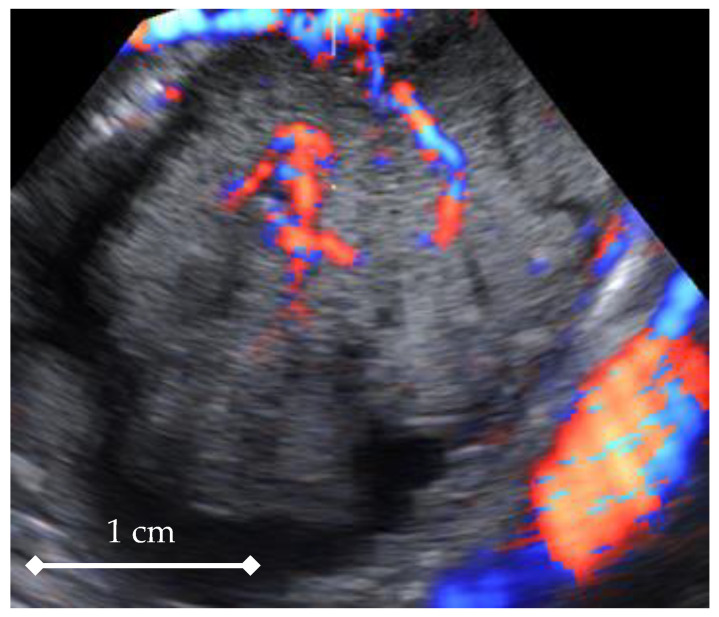
STIC 4D sequence point from an ovarian tumor (highest vascularization region), depicting the systolic moment of the cardiac cycle.

**Figure 3:**
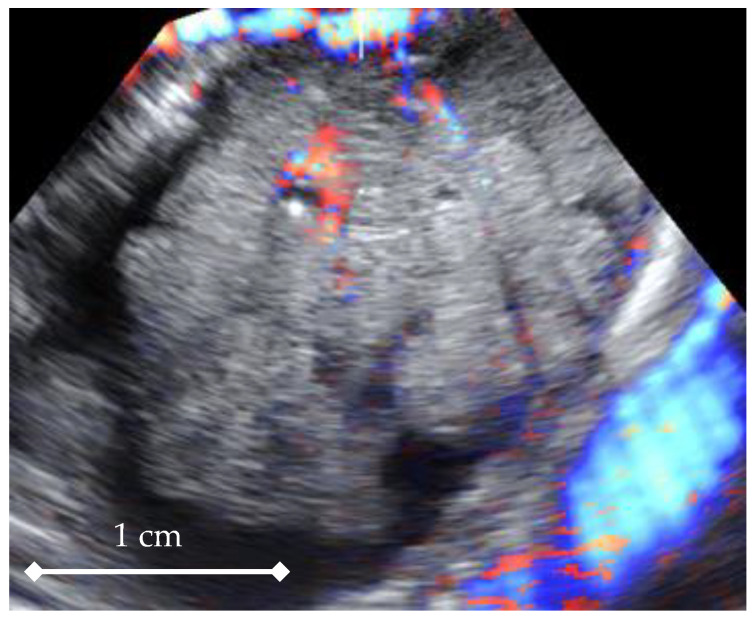
STIC 4D sequence point from an ovarian tumor (highest vascularization region), depicting the diastolic moment of the cardiac cycle.

**Figure 4:**
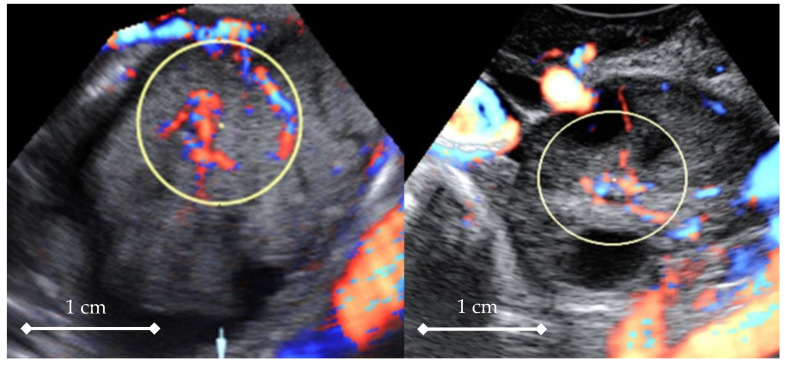
Ovarian tumor—an example of virtual spherical sampling of the most vascularized part of the tumor tissue—systole (**left**); and normal ovary—systole (**right**).

In the next step, all 3D volumes were consecutively activated, and with 4D-View software, the center of a 1cc virtual sphere was placed in the center of the displayed picture, which already represented the point of highest ovarian/tumor tissue vascularization during the systole and diastole of the same heart cycle (Figure 2 and Figure 3). The 1cc spherical volume (tolerance limit ± 0.05cc) was set up semi-automatically by the operator (Figure 4).

After the sphere size was accepted for each volume in turn, HDF histograms including 3D indices were automatically calculated based on which two 3D volumes were picked, with their VI values being, respectively, the highest for systole and the lowest for diastole. In such a way, 3D Vascularization Index (VI) values were obtained from the same ovarian/tumor region at two different moments during the cardiac cycle: systolic VI (VIsys) and diastolic VI (VIdias) (Figure 2 and Figure 3, Supplementary Video S1). The mean Vascularization Index (VI) value was calculated based on the results from all volumes.

Using these 3D VI values, two 4D indices were calculated with the following formulas [12]:

volumetric Pulsatility Index (vPI) = (systolic VI − diastolic VI)/mean VI

volumetric Systolic/Diastolic Index (vS/D) = systolic VI/diastolic VI

The same examiner (M.J.K.) performed all STIC-HDF volume acquisitions and analyses.

The Kolmogorov–Smirnov test was used to assess normal distribution of continuous variables (age, VIsys, VIdias, vPI, and vS/D). Data are expressed as mean with standard deviation (SD) or median with interquartile range (IQR), depending on whether the distribution is normal or not. Categorical variables are presented as number and percentage.

One-way analysis of variance (ANOVA) or U Mann–Whitney tests were used to compare mean or median values, respectively. A *p*-value < 0.05 was considered as statistically significant. SPSS 20.0 statistical software was used for all calculations.

## 3. Results

During the study period, 40 cases of ovarian tumors were recruited. Eighteen cases were excluded for the following reasons: no histology (n = 3), malignancies other than HGSC (n = 9), and non-malignant histology (n = 6).

Finally, 4D STIC data derived from a group of 23 HGSC cases and 23 normal ovaries were analyzed. Assessable 4D-STIC sequences were obtained in every study and control case. The median age in the study group (57.5 years (SD: 16.7), range: 27 to 86) was similar to the control group (57.1 years (SD: 16.1), range: 30 to 83) (*p* > 0.05).

**Figure 5:**
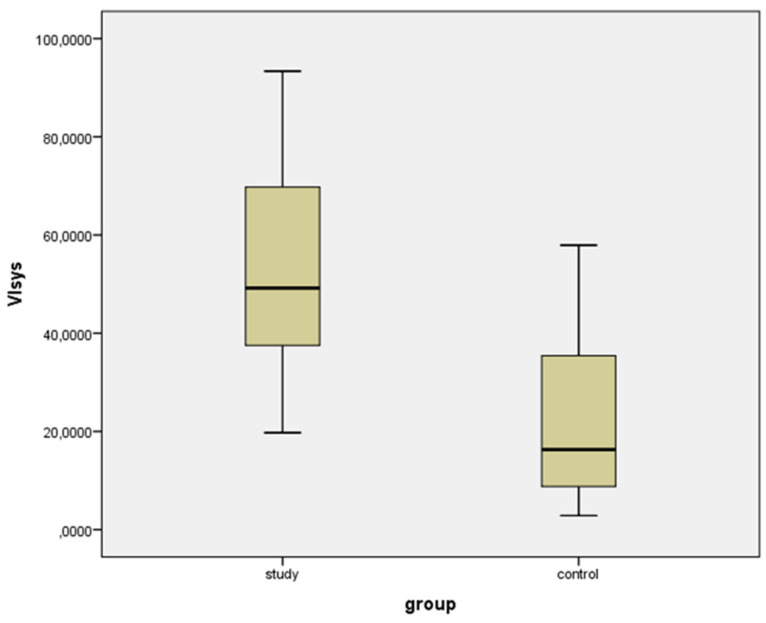
Box-plot for Vascularization Index (3D) in systole (VIsys) for study and control group.

**Figure 6:**
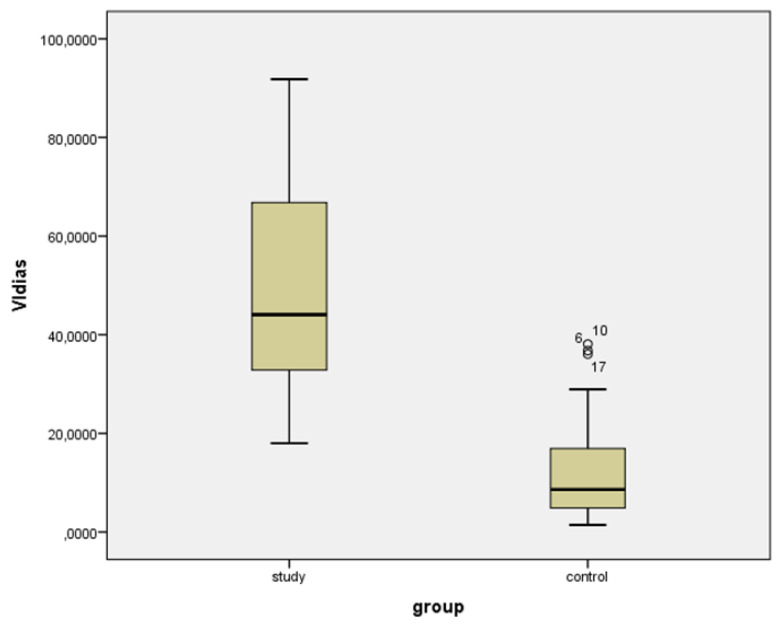
Box-plot for Vascularization Index (3D) in diastole (VIdias) for study and control group.

Median values for VIsys and VIdias were significantly higher in the study group as compared to the control group (Table 1) (Figure 5 and Figure 6).

**Figure 7:**
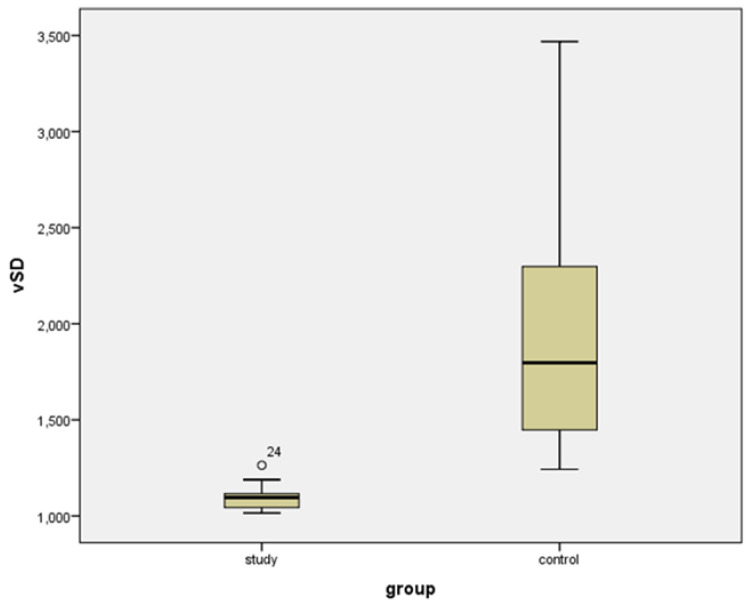
Box-plot for volumetric (4D) Systolic/Diastolic (vS/D) Index for study and control group.

**Figure 8:**
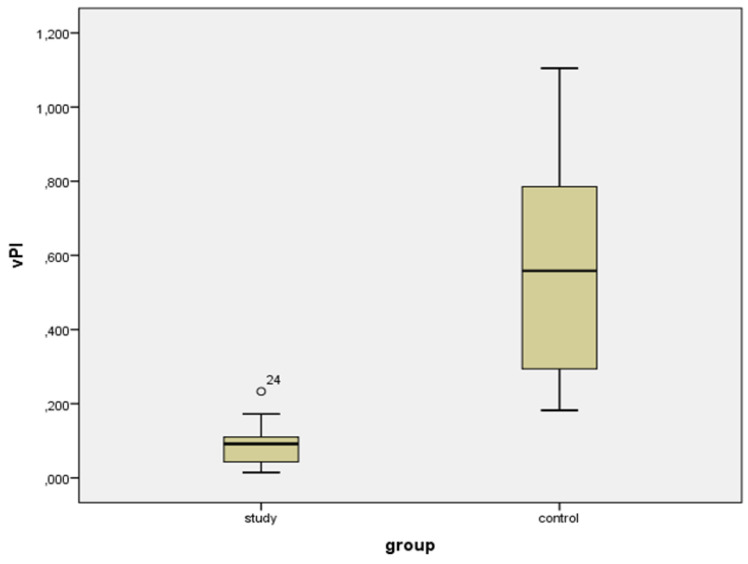
Box-plot for volumetric (4D) Pulsatility Index (vPI) for study and control group.

Values of volumetric S/D indices and volumetric PI indices were statistically lower in ovarian tumors compared to normal ovaries: 1.096 vs. 1.794 and 0.092 vs. 0.558, respectively (*p* < 0.001) (Table 1) (Figure 7 and Figure 8).

## 4. Discussion

Since the introduction of the Doppler technique as a complement to the 2D ultrasound evaluation, attempts have been made to use its results to improve the sensitivity and specificity of the method. Initially, mainly color Doppler was used to assess tissue vascularity (qualitative assessment) and the spectral Doppler option was used to assess the flow velocity (quantitative assessment).

**Figure 9:**
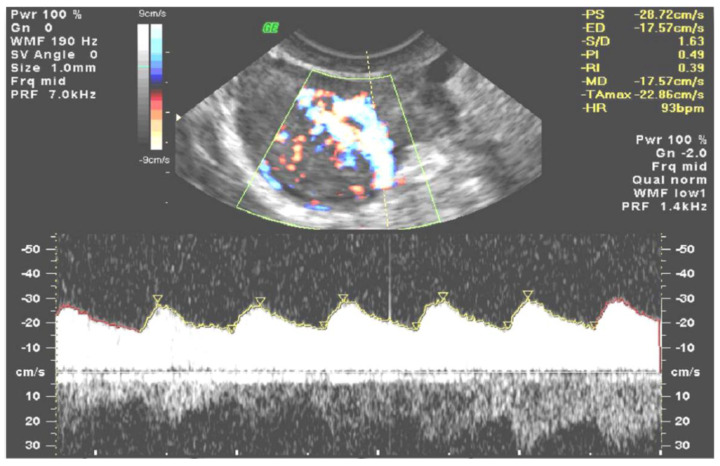
Spectral Doppler. Assessment of the blood flow in a single tumor vessel. The results of calculated 2D flow indices (S/D, PI, and RI) can be seen in the upper right corner.

It soon turned out that the assessments of the flow velocities themselves were very ambiguous. To improve the repeatability of the method and the possibility of comparing the results of various tests, the so-called 2D flow indices, i.e., Systolic/Diastolic Index, Resistance Index, and Pulse Index, were introduced. These indices are still in use today in the assessment of adnexal tumors in gynecology (Figure 9), where, on the basis of blood velocity spectrum, they reflect differences between vessel wall structures of neoplastic tumors and vessels of normal tissues. In the cases of neoplastic tumors, the walls of their pathological, rapidly growing vessels are less flexible than in normal tissue, which causes the difference in blood velocity during systole and diastole to decrease, and this results in “flattening” the velocity graph during one heart cycle. This results in lower S/D and PI scores for tumor vessel flow. A number of scientific studies using these observations have been already published [16]; however, this method has very important limitations. The main problem is that observation is limited to a single “main tumor vessel” only. This does not take into account the flow throughout the whole tumor, but is rather based on the subjective choice of the operator.

The next development of qualitative Doppler evaluation techniques was the introduction of the power Doppler 2D option, which is less angle dependent and much more sensitive to flows in small, pathological tumor vessels because it relies on vascular flow energy instead of velocity. This technique was introduced as a qualitative rather than quantitative method for 2D imaging studies.

**Figure 10:**
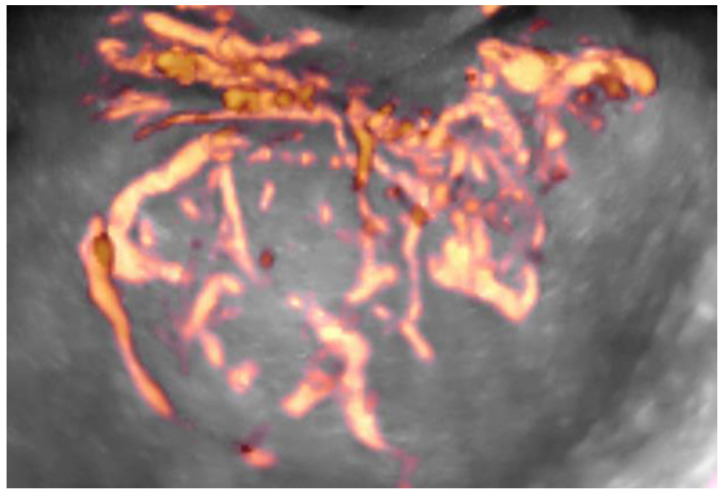
3D Power Doppler spatial image of the net of vessels (yellow-red color areas) supplying an ovarian tumor (Glass Body option).

The introduction of 3D imaging options fundamentally changed this situation and allowed the use of Power Doppler as a quantitative method. The 3D Power Doppler ultrasound technique allows for the visualization of the spatial network of vessels supplying the entire organ (Figure 10, Supplementary Video S2).

**Figure 11:**
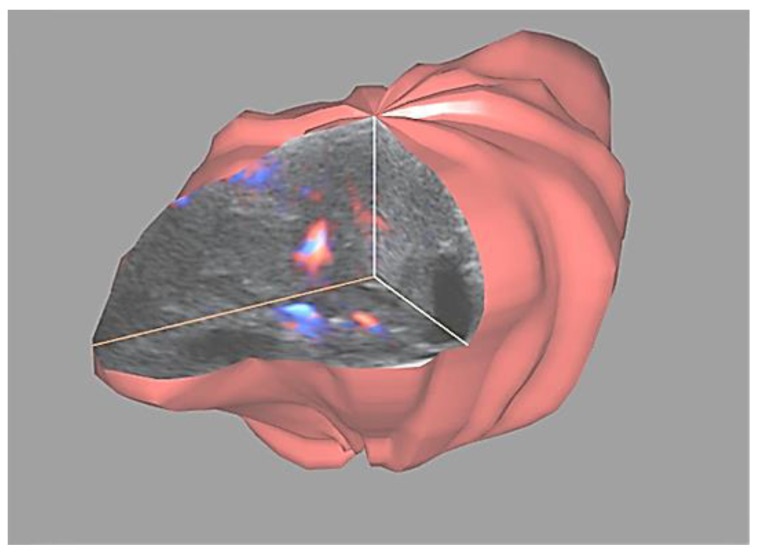
Virtual shell corresponding to the ovarian tumor in a 3D visualization with visible vessels inside the tumor (High Definition Flow). Vascularization Index value calculated on this basis was equal to 13.

After visualizing this network, by virtually cutting out the volume of interest, we can calculate the 3D Vascularization Index (VI), which reflects the percentage of the volume occupied by the vessels within the volume of interest (Figure 11, Supplementary Video S3).

**Figure 12:**
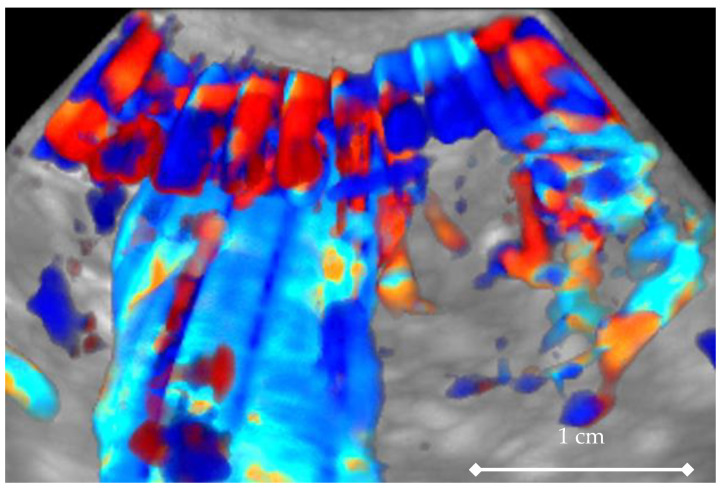
Color Doppler 3D magnified image of the transverse vessel (upper part of the image). The influence of periodically variable (systolic/diastolic) blood flow is visible as linear vertical irregularities in color. Creating a single record with ultrasound beam moving from left to right of the screen takes about 10s which covers about 10 heartbeat cycles.

The value of this index is expressed as a percentage (from 0 to 100). This information may be valuable in the context of the known fact that cancerous and rapidly growing lesions usually have denser and more intense blood supply, which results in higher values of VI [17,18]. The problem, however, is the lack of standardization of this method; therefore, its utility is limited [5,6,19]. One of the main limitations here is that the visualization of the vessels is strongly dependent on the settings of ultrasound system being used, and on the distance between the tumor and the active surface of the ultrasound transducer. This affects the final numerical value of the VI index, which may differ significantly between two consecutive assessments of the same tumor. The second important issue limiting 3D Doppler ultrasound is the fact that collecting the ultrasound reflected signals for one 3D record takes up to about 10 s. This means that an ultrasound beam passing, for example, 90 degrees in the space filled by the vessels, will collect information sequentially, during systole and diastole. Consequently, this means that some areas will be visible with prominent vessels while others will be less visible (Figure 12). Thus, the value of the 3D Vascularization Index will represent the average value of the blood supply depending also on the moment of the heart cycle in which we start to obtain the record. It can influence the final value of the VI.

In contrast, the 4D record, which consists of a sequence of 3D images (so called “volumes”) representing different phases of blood supply during one heart cycle, allows for the precise selection of the moment of the highest and the lowest blood supply to a given organ (Figure 1, Supplementary Video S4). This standardizes and makes the assessment of the 3D blood supply independent from the starting moment of the record, because the value of VI will be precisely determined by the average of the maximum and minimum values ((VImax + VImin)/2).

In addition, the main benefit of a 4D exam is an indirect assessment of the futures of the vessels in given volume—the same way it was done with the 2D spectral Doppler method. As pathological tumor vessels are less elastic than normal vessels, the S/D index (which is a quotient of maximum blood velocity divided by minimum velocity value) is lower.

The 4D technique allows us to use this concept by extending evaluation to all tumor vessels in its volume and replacing S/D and PI indices (2D) by use of volumetric vS/D and vPI indices (4D).

The concept explained above was the basis of the presented research work. Isolating blood supply (VI values) from two phases—maximum (systolic VI) and minimum (diastolic VI)—allowed us to increase the objectivity of the method by calculating the quotient vS/D = VIsys/VIdias, similar to the Spectral Doppler technique, where we do not measure the individual maximum or minimum flow velocities, but rather calculate the maximum to minimum ratio (S/D ratio). Thus, according to our study, the properties of the pathological vascular network of neoplastic tumors and normal ovaries were compared, with the use of 4D Doppler ultrasound.

Research in 4D angiography in gynecology is still scarce. The first study on that subject was published in 2010 [20]. Subsequent papers allowed for better understanding of the method as well as the development of new vascular indices similar to those used in spectral Doppler 2D [11,12]. The observations made so far were related mainly to laboratory tests [21,22,23,24,25]. Bidirectional Power Doppler (HDF) was decided to be the option used in vessel net detection. This technique seemed to have better axial resolution, fewer blooming artifacts, and improved sensitivity to small vessels compared to Power Doppler and Color Doppler [26]. The abovementioned technology had already been applied in clinical trials for assessment of vascularization in evaluation of endometrium [12] and polycystic (PCOS) ovaries [20].

The other factor limiting our ability to use 3D/4D quantitative methods of vascularization analysis is the proper and repeatable volume size. Quantitative evaluations of the same region in the same organ with different volume sizes will render different numerical results [27]. This is why a fixed sample volume was decided to be used in this study for the analysis of blood supply. The 1cc spherical sample was decided to be applied because of the limitation of the ovary size in postmenopausal women within the control group (Figure 4, Supplementary Video S1). Ovaries in these women are too small for a larger size sphere to be wholly contained in them. Technically, the most representative vascularized part of the tumor was selected and 1cc spherical samples were taken from it (Supplementary Video S1). There were two important reasons for doing so. One is the technical limitation of up-to-date technology that limits the volume that can be examined in a single scan by 3D/4D transducers. This fact was already recognized in previous papers dealing with larger structures like uterine malformations, fibroids, and ovarian tumors [2,5]. On top of that, distance should be taken into account, since Doppler signals are strongly attenuated, and this may significantly affect the values of Indices obtained by 3D/4D Doppler [28]. This means that only the first few centimeters from the transducer’s active face can be considered as representing reliable Doppler information for this kind of technique. This forces us to limit the size of the volume and to visualize smaller parts of the structure.

Our study has some limitations. First, the sample size is small and we did not perform power calculations, so we cannot ascertain how reliable our results are. Second, a reproducibility assessment was not performed. Therefore, we do not know how reproducible this technique is. Third, we did not compare malignant tumors with benign ovarian tumors but with normal ovaries. Fourth, the study focused on HGSC only. This type of cancer is the most frequent ovarian cancer and the most aggressive. This is the reason why we focused on it. Nevertheless, clearly there is a selection bias.

When considering the assessment of blood supply and the differences between nets of blood vessels supplying normal and cancerous ovaries, one should also take into account the fact of menopausal changes influencing physiological reduction of their elasticity [29,30]. This issue is important, as many ovarian cancer patients are postmenopausal. Normal ovarian vessels are stiff; however, during menopause, they even increase stiffness. This results in increasing systolic-diastolic difference in blood flow. On the other hand, newly emerging pathological neoplastic vessels of a tumor are devoid of the muscularis, which makes them more susceptible and reduces the systolic-diastolic difference in flow in them. These differences between nets of supplying vessels that cannot be detected by 3D (static) technology when evaluated by 4D vascularity index values should be even more pronounced, improving the ability to distinguish physiology from pathology.

In summary, the results of the study showed that the dynamic assessment of blood supply changes during one heart rate, expressed with 4D indices (Volumetric Pulsatility Index and Volumetric Systolic/Diastolic Index), can be a method of differentiating between normal and pathological ovaries as 4D bi-directional Power Doppler displayed statistically significant vascularization differences between normal and malignant ovaries. Indices were lower when derived from malignant tissue vessels. Additionally, the advantage of this method is the fact that the 4D blood flow rates are mathematically the quotient of 3D vascularization values. This means that they are much less exposed to the influence of the ultrasound system settings and tissue properties compared to the 3D technique. Moreover, this method indirectly allows for the assessment of the elasticity of the vessel wall and possibly could replace the contrast methods for the assessment of blood supply changes.

The main strength of our study is that to the best of our knowledge, no single study has been reported assessing whether the values of these new 4D vascular indices are different when derived from vessels in cancer tissue as compared to those derived from vessels from normal tissue. In spite of the above-mentioned limitations, we think that this data is encouraging and would support future multi-center research into the use of 4D Power Doppler.

## Figures and Tables

**Table 1:** Vascularization (3D) index in systole (VIsys) and diastole (VIdias) and volumetric 4D indices vS/D and vPI, in study and control group.

	VIsys *		VIdias *		vS/D ^+^		vPI ^+^	
	Mean (%) (SD)	Range	Mean (%) (SD)	Range	Median (IQR)	Range	Median (IQR)	Range
Study group	53.729 (22.104)	19.737 to 93.359	49.169 (21.827)	18.508 to 91.800	1.096 (0.770)	1.015 to 1.204	0.092 (0.071)	0.015 to 0.233
Controls	22.201 (15.989)	2.876 to 57.912	13.447 (11.835)	1.440 to 38.116	1.794 (0.994)	1.243 to 3.469	0.558 (0.581)	0.182 to 1.105

* Analysis of variance (ANOVA), *p* < 0.0001. ^+^ U Mann–Whitney, *p* < 0.0001. SD: Standard deviation. IQR: Interquartile range.

## Data Availability

The data analyzed in the study is available from the corresponding author on reasonable request.

## Supplementary Materials

The following are available online at https://www.mdpi.com/2075-4418/11/4/582/s1.
